# Association of BDNF and BMPR1A with clinicopathologic parameters in benign and malignant gallbladder lesions

**DOI:** 10.1186/1477-7819-11-80

**Published:** 2013-03-26

**Authors:** Li Xiong, Xiaofeng Deng, Yu Wen, Zhulin Yang, Xiongying Miao

**Affiliations:** 1 Department of General Surgery, Second Xiangya Hospital, Central South University, Changsha, Hunan 410011, PR China; 2 Research Laboratory of Hepatobiliary Diseases, Second Xiangya Hospital, Central South University, Changsha, Hunan 410011, PR China

**Keywords:** Gallbladder lesions, BDNF, BMPR1A, Clinicopathology

## Abstract

**Background:**

Neurotrophic factors such as brain derived neurotrophic factor (BDNF) are synthesized in a variety of neural and non-neuronal cell types and regulate survival, proliferation and apoptosis. In addition, bone morphogenetic proteins (BMPs) inhibit the proliferation of pulmonary large carcinoma cells bone morphogenetic protein receptor, type IA (BMPR1A). Little is known about the expression of BDNF or BMPR1A in malignant gall bladder lesions. This study was to evaluate BDNF and BMPR1A expression and evaluate the clinicopathological significance in benign and malignant lesions of the gallbladder.

**Methods:**

The BDNF and BMPR1A expression of gallbladder adenocarcinoma, peritumoral tissues, adenoma, polyp and chronic cholecystitis were Immunohistochemically determined.

**Results:**

BDNF expression was significantly higher in gallbladder adenocarcinoma than in peritumoral tissues, adenoma, polyps and chronic cholecystitis samples. However, BMPR1A expression was significantly lower in gallbladder adenocarcinoma than in peritumoral tissues, adenomas, polyps and chronic cholecystitis tissues. The specimens with increased expression of BDNF in the benign lesions exhibited moderate- or severe-dysplasia of gallbladder epithelium. BDNF expression was significantly lower in well-differentiated adenocarcinomas with maximum tumor diameter <2 cm, no metastasis to lymph nodes, and no invasion of regional tissues compared to poorly-differentiated adenocarcinomas with maximal tumor diameter >2 cm, metastasis of lymph node, and invasiveness of regional tissues in gallbladder adenocarcinoma. BMPR1A expression were significantly higher in the well-differentiated adenocarcinoma with maximal tumor diameter <2 cm, no metastasis of lymph node, and no invasion of regional tissues compared to poorly-differentiated adenocarcinomas with maximal tumor diameter >2 cm, metastasis of lymph node, and invasiveness of regional tissues in gallbladder. Univariate Kaplan-Meier analysis indicated increased expression of BDNF or decreased expression of BMPR1A was associated with decreased disease specific survival (DSS) rates. Similarly, multivariate Cox regression analysis showed increased expression of BDNF or decreased expression of BMPR1A are independent predictors of poor DSS rates in gallbladder adenocarcinoma.

**Conclusions:**

In gallbladder malignancies, the increased expression of BDNF and decreased expression of BMPR1A were associated with increased risk of metastasis, regional invasion and mortality. They might serve as novel indicators of gallbladder adenocarcinoma outcomes, which may prove valuable for the development of personalized therapeutic paradigms.

## Background

Gallbladder cancer is the most common biliary tract malignancy and the fifth most common malignant neoplasm of the digestive tract [1]. Despite recent advances in the diagnosis and management of gastrointestinal cancers, cancer of the gallbladder remains a neoplasm with a high mortality rate and poor overall prognosis. More than 85% of gallbladder cancers are adenocarcinomas that are well or moderately differentiated, while the remaining 15% are squamous, adenosquamous, or undifferentiated carcinoma [2]. Many of these tumors are not resectable at the time of presentation because of local invasion of crucial gallbladder structures, or metastasis into surrounding tissues and lymph nodes. The primary TMN (tumor, node, meta stasis’ age is a significant prognostic factor, and the 5-year survival rate for patients with T3 or T4 carcinoma has been reported as less than 15% [3]. Therefore, better understanding of the pathogenesis of gallbladder cancer, including the expression of tumor-specific markers, should provide a basis for developing novel therapies [4].

Brain-derived neurotrophic factor (BDNF), a secreted protein, is a member of the neurotrophin growth factor family. BDNF is expressed by several human tumors, and is a key regulator of oncogenesis, tumor invasion, and tumor progression. BDNF knockdown may inhibit tumor invasion of HepG2 and HCCLM3 cells [5,6]. Recent studies have shown that high expression levels of BDNF have a significant relationship with the tumorigenesis, progression, biological behavior, and prognosis of breast cancers [7,8]. Furthermore, Artico *et al*. recently reported that increased expression of BDNF was clearly visible in gallbladder carcinoma, suggesting a direct role of this neurotrophic factor in the transformation and progression of neoplastic cells of gallbladder tissue. However, whether increased expression of BDNF is associated with disease prognosis remains unknown.

Bone morphogenetic proteins (BMPs) are a group of multifunctional growth factors of the transforming growth factor (TGF)-β superfamily of cytokines [9,10]. Numerous studies have shown that these morphogens play crucial roles during development and in the regulation of cell proliferation, differentiation, and apoptosis [11]. BMPs exert their biological effects through bone morphogenetic protein receptor type 1A (BMPR1A), also known as ALK3, which is a transmembrane serine/threonine kinase expressed in several tissues and cancers [12]. BMPR1A is a type I receptor of the TGF-β superfamily, with a cysteine-rich extracellular region, an intracellular glycine–serine-rich (GS) domain near the plasma membrane, and an intracellular kinase domainBMPR1A mediates BMP intracellular signaling, which is associated with carcinogenesis, through MADH4 [13,14]. Recent studies have reported that low BMPR1A expression in tumors may be associated with poor prognosis [15,16]. However, the expression levels of BDNF and BMPR1A and their clinicopathologic significance in malignant tumors, particularly gallbladder cancer, have not been thoroughly evaluated.

## Methods

### Ethics approval

This study was approved by the institutional ethics committee of Second Xiangya Hospital, Central South University, and performed in accordance with the Declaration of Helsinki (2000) of the World Medical Association. All patients provided written informed consent.

### Specimens and clinicopathologic material

Surgically resected or biopsy specimens were collected from Xiangya Hospital (Central South University, Hunan, PR China), between 1996 and 2011. Adenoma specimens were collected from 100 patients with gallbladder adenocarcinoma (70 women, 30 men; mean age 52.4 ± 11.3 years). In addition, 46 peritumoral tissues were harvested from these 100 adenocarcinomas, and we also obtained 15 gallbladder polyp tissues, 30 gallbladder adenoma tissues, and 35 chronic cholecystitis tissues from other patients. All diagnoses were based on morphological criteria, immunohistochemical staining, and clinical findings. The histopathological subtypes of the 100 adenocarcinoma specimens were: 36 well-differentiated adenocarcinomas, 29 moderately differentiated adenocarcinomas, 25 poorly differentiated adenocarcinomas, and 10 mucinous adenocarcinomas. Of the 100 patients with adenocarcinoma, 54 had invasion of surrounding tissues and organs, 59 had regional lymph-node metastasis, and 58 had gallstones. According to the standard criteria for T stages [17], of the 100 adenocarcinomas, 22 were at stage T1, 35 at T2, 29 at T3, and 14 at T4. Radical resections were performed in 32 of the adenocarcinoma patients, palliative surgery in 46, and biopsy in the remaining 22 patients. Follow-up was completed for 65 of the 100 patients by letters and phone calls; these data showed that 20 patients survived over 1 year, and 45 patients survived for less than 1 year.

Of the 46 peritumoral tissues (distance from cancer ≥ 3 mm), 10 were classified as normal, while 10 had mild dysplasia, 12 had moderate dysplasia, and 14 had severe dysplasia. The diameters of the 15 gallbladder polyps ranged from 8 to 15 mm. Ten of the cases were pathologically classified as having normal epithelium to mild dysplasia, and five cases as having moderate to severe dysplasia. The diameter of the 30 gallbladder adenomas was between 9 to 22 mm. There were five cases pathologically confirmed as normal mucosa, ten as mild dysplasia, nine as moderate dysplasia, and six as severe dysplasia. Of the 35 patients with chronic cholecystitis, 15 had chronic cholecystitis alone, whereas the other 20 patients had chronic cholecystitis accompanied by gallstones. The pathological examination confirmed that 11 of the gallbladders had normal mucosa, 12 had mild dysplasia, 7 had moderate dysplasia, and 5 had severe dysplasia.

### Immunohistochemistry

Sections 4 μm thick were cut from routinely processed tissues that had been embedded in paraffin wax. Immunohistochemical staining was carried out using rabbit anti-human BDNF and BMPR1A polyclonal antibodies (gift from Abgent Company, San Diego CA, USA) and a commercial kit (EnVision^TM^ Detection Kit; Dako Laboratories, Carpinteria, CA, USA) in accordance with the manufacturer’s protocol (ChemMate^TM^ EnVision+/HRP/DAB).

Briefly, the sections were dewaxed in distilled water, and incubated with peroxidase inhibitor (3% H_2_O_2_) in the dark for 15 minutes, then washed with distilled water, followed by EDTA-trypsin digestion (0.125%, pH 9.0) for 15 minutes, and further washing with distilled water. After incubating the sections three times in phosphate-buffered saline (PBS, pH 7.4) for 5 minutes each the sections were removed and the liquid around the tissues removed, without drying the tissues themselves. The sections were then incubated with rabbit anti-BDNF or anti-BMPR1A for 60 minutes at room temperature. The sections were socked in PBS three times for 5 minutes each, then the sections were removed, and the liquid wiped from around the tissues as before. The first solution from the kit (solution A) was added to the sections, which were incubated for 30 minutes at room temperature, then the sections were removed, and the liquid wiped from around the tissues as before. Diaminobenzidine substrate was added to the sections and left for of 5 to 10 minutes at room temperature. After staining was complete, the sections were washed with distilled water, then counterstained with hematoxylin for 1 minute, and incubated in distilled water for 15 minutes. The slides were dehydrated with increasing concentrations (70 to 100%) of alcohol, each for 3 minutes. The sections were incubated in xylene three times for 5 minutes, and finally mounted on slides with neutral balsam.

Cells in the cytoplasm and cell membrane containing brown-yellow granules were defined as positive cells. The percentage of positive BDNF or BMPR1A cells was calculated from 10 random high fields. The sample was considered positive when the percentage of stained cells was 25% or higher, and considered negative when the percentage of stained was less than 25%.

### Statistical analysis

The data were analyzed using SPSS software (version 13.0; SPSS Inc., Chicago, IL, USA). The inter-relationship of BDNF or BMPR1A expression with the histological or clinical factors was analyzed by the independent χ^2^ test. Fisher’s exact probability test was used to analyze the statistical association between the two independent sample groups. Kaplan–Meier and log-rank tests were used for univariate survival analysis. The Cox proportional hazards model was used for multivariate analysis and to determine the 95% confidence interval.

## Results

### Expression levels of brain-derived neurotrophic factor and bone morphogenetic protein receptor type IA in benign and malignant gallbladder lesions

**Figure 1:**
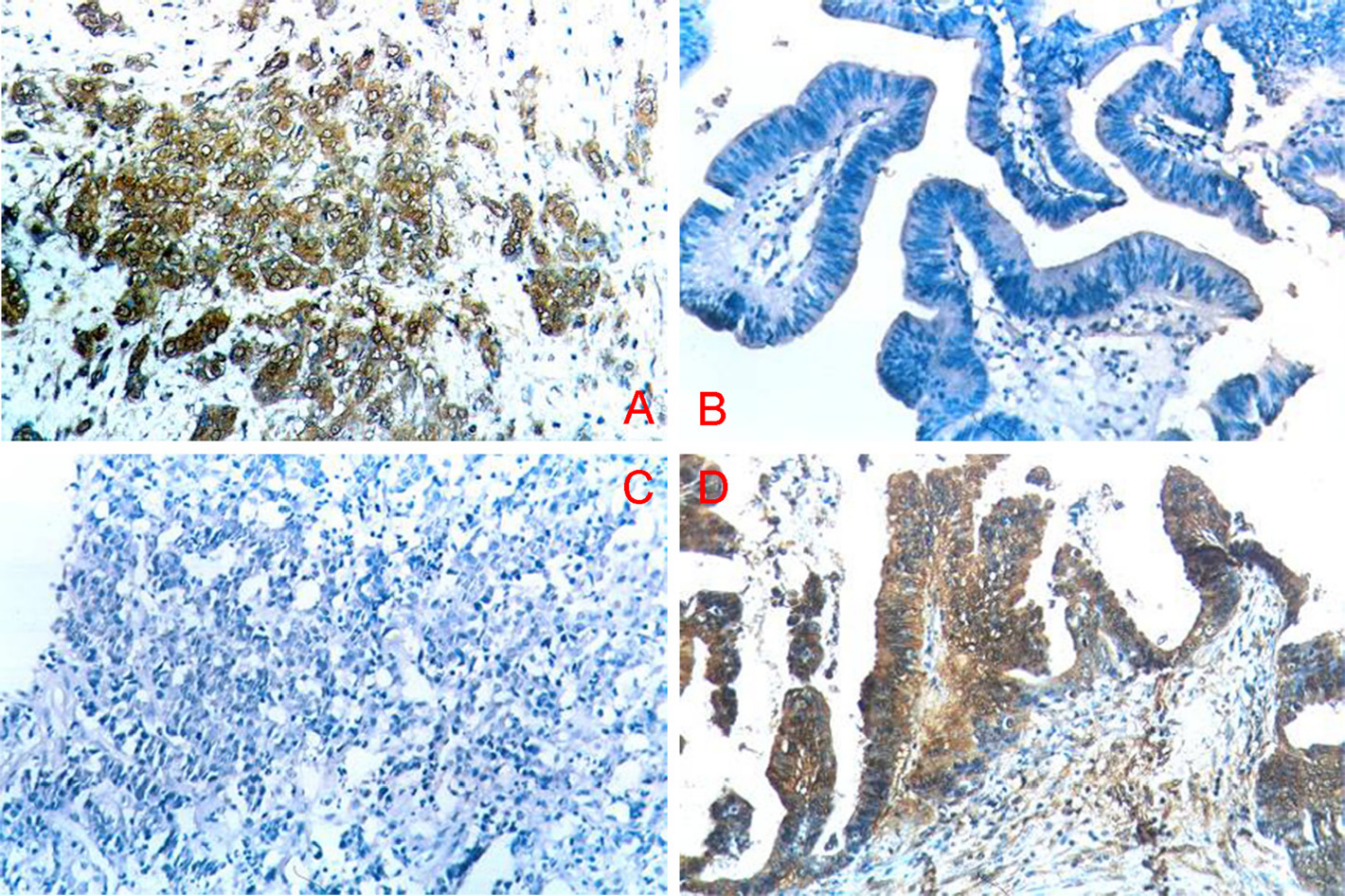
**Expression of brain-derived neurotrophic factor (BDNF) and bone morphogenetic protein receptor type IA (BMPR1A)in benign and malignant gallbladder lesions. (A)** Positive expression of BDNF in adenocarcinoma, and **(B)** negative expression of BDNF in a benign lesion. **(C)** Negative expression of BMPR1A in adenocarcinoma and **(D)** positive expression of BMPR1A in a benign lesion. The positive reaction for both BDNF and BMPR1A was mainly localized to the cytoplasm and/or cell membrane. Original magnification × 200.

**Figure 2:**
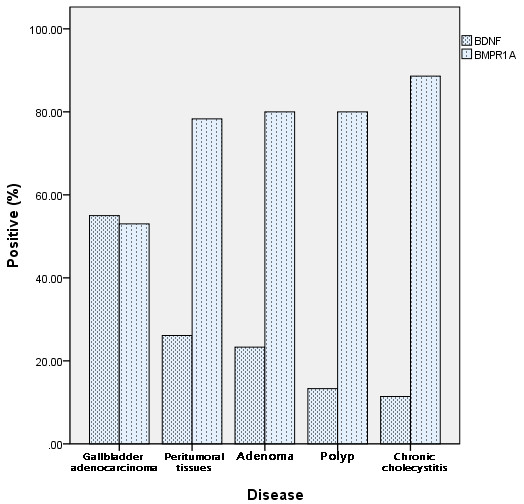
Expression of brain-derived neurotrophic factor (BDNF) and bone morphogenetic protein receptor, type IA (BMPR1A) in gallbladder adenocarcinoma, peritumoral, adenoma, polyp, and chronic cholecystitis tissues.

Positive immunohistochemical staining for BDNF and BMPR1A was seen in the cytoplasm and/or on the cell membrane (Figure 1A–D). BDNF was significantly higher (positive) and BMPR1A was significantly lower (negative) in gallbladder adenocarcinoma than in peritumoral tissues, adenoma, polyps, and chronic cholecystitis (*P* < 0.01 for both) (Table 1, Figure 2). In addition, the BDNF positivity and BMPR1A negativity in peritumoral tissues, polyps, and chronic cholecystitis epithelium was accompanied by moderate or severe dysplasia. When all100 gallbladder adenocarcinoma, 46 peritumoral, 30 adenoma, 15 polyp and 35 chronic cholecystitis samples were assessed, the positive rate of BDNF was inversely related to that of BMPR1A, due to the analysis of Pearson correlation coefficients (r = −7.482, *P* < 0.05).

**Table 1:** Expression of brain-derived neurotrophic factor (BDNF) and bone morphogenetic protein receptor type IA (BMPR1A) in gallbladder adenocarcinoma, peritumoral, adenoma, polyp, and chronic cholecystitis tissues

**Tissue**	**Total, n**	**BDNF**			**BMPR1A**		
		**Positive, n (%)**	***χ***^***2***^	***P***	**Positive, n (%)**	***χ***^***2***^	***P***
Gallbladder adenocarcinoma	100	55 (55.0)			53 (53.0)		
Peritumoral tissues	46	12 (26.1)	10.61	<0.01	36 (78.3)	8.45	<0.01
Adenoma	30	7 (23.3)	9.28	<0.01	24 (80.0)	6.97	<0.01
Polyp	15	2 (13.3)	9.06	<0.01	12 (80.0)	3.87	<0.01
Chronic cholecystitis	35	4 (11.4)	20.01	<0.01	31 (88.6)	13.96	<0.01

### Expression of brain-derived neurotrophic factor and bone morphogenetic protein receptor type IA and evaluation of correlation with the clinicopathologic features of gallbladder adenocarcinoma

**Figure 3:**
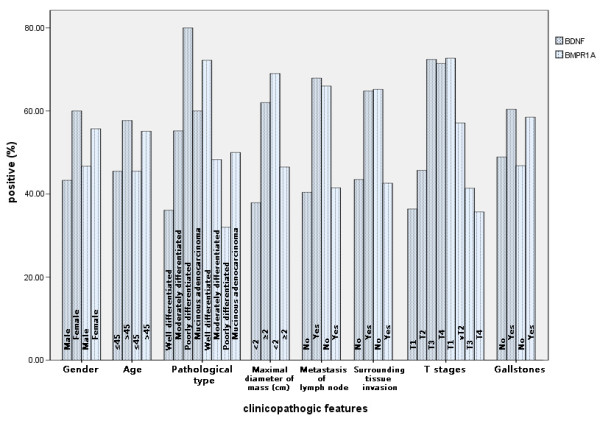
Expression of brain-derived neurotrophic factor (BDNF) and bone morphogenetic protein receptor type IA (BMPR1A) and their correlation with the clinicopathologic parameters of gallbladder adenocarcinoma.

BDNF expression was significantly lower (*P* < 0.05) and BMPR1A was significantly higher (*P* < 0.05) in the well-differentiated gallbladder adenocarcinomas with maximum diameter of <20 mm, no metastasis to lymph nodes, and no invasion of regional tissues, compared with poorly differentiated tissues with maximal mass diameter of >20 mm, metastasis to lymph nodes, and invasion of regional tissues. There was no correlation between expression of BDNF or BMPR1A and age, gender, or presence or absence of gallstones (*P* > 0.05) (Table 2, Figure 3).

**Table 2:** Expression of brain-derived neurotrophic factor (BDNF) and bone morphogenetic protein receptor type IA (BMPR1A) and their correlation with the clinicopathologic parameters of gallbladder adenocarcinoma

**Clinicopathologic features**	**Cases**	**BDNF**			**BMPR1A**		
		**Positive cases, n (%)**	***χ***^***2***^	***P***	**Positive cases, n (%)**	***χ***^***2***^	***P***
Gender							
Male	30	13 (43.3)	2.36	>0.05	14 (46.7)	0.69	>0.05
Female	70	42 (60.0)			39 (55.7)		
Age, years							
≤ 45	22	10 (45.5)	1.04	>0.05	10 (45.5)	0.65	>0.05
> 45	78	45 (57.7)			43 (55.1)		
Pathological type^a,b,c^							
Well differentiated	36	13 (36.1)	11.60	<0.05	26 (72.2)	12.40	<0.05
Moderately differentiated	29	16 (55.2)			14 (48.3)		
Poorly differentiated	25	20 (80.0)			8 (32.0)		
Mucinous adenocarcinoma	10	6 (60.0)			5 (50.0)		
Maximum diameter of mass, mm							
< 20	29	11 (37.9)	4.81	<0.05	20 (69.0)	4.18	<0.05
≥ 20	71	44 (62.0)			33 (46.5)		
Metastasis to lymph node							
No	47	19 (40.4)	7.61	<0.01	31 (66.0)	5.98	<0.05
Yes	53	36 (67.9)			22 (41.5)		
Invasion to surrounding tissue							
No	46	20 (43.5)	4.57	<0.05	30 (65.2)	5.10	<0.05
Yes	54	35 (64.8)			23 (42.6)		
T stage							
T1	22	8 (36.4)	8.28	<0.05	16 (72.7)	11.79	<0.05
T2	35	16 (45.7)			20 (57.1)		
T3	29	21 (72.4)			12 (41.4)		
T4	14	10 (71.4)			5 (35.7)		
Gallstones							
No	47	23 (48.9)	1.32	>0.05	22 (46.8)	1.37	>0.05
Yes	53	32 (60.4)			31 (58.5)		

^a^Well-differentiated compared with moderately differentiated for BDNF: *χ* = 2.36, *P* > 0.05; and for BMPR1A: *χ*^*2*^ = 1.56, *P* > 0.05.

^b^Well-differentiated compared with poorly differentiated for BDNF: *χ*^*2*^ = 11.44, *P* < 0.01; and for BMPR1A: *χ*^*2*^ = 9.68, *P* < 0.01.

^c^Moderately differentiated compared with poorly differentiated for BDNF: *χ*^*2*^ = 3.72, *P* = 0.05; and for BMPR1A: *χ*^*2*^ = 1.47, *P* > 0.05.

### Correlation of brain-derived neurotrophic factor and bone morphogenetic protein receptor type IA expression with disease-specific survival of gallbladder adenocarcinoma

Follow-up at year5 was carried out by phone or mail surveys, and data were collected from 65of the 100 patients. Of these 65 patients, 20 had a survival of over 1 year, and the remaining 45 died within 1 year after surgery as a result of tumor progression, with a mean survival time of 9.7 ± 5.4 months. Positive immunohistochemical staining for BDNF was found for 32 (49.2%) of the 65 patients, and positive staining for BMPR1A was found for 31 (47.7%) of the 65 patients.

**Figure 4:**
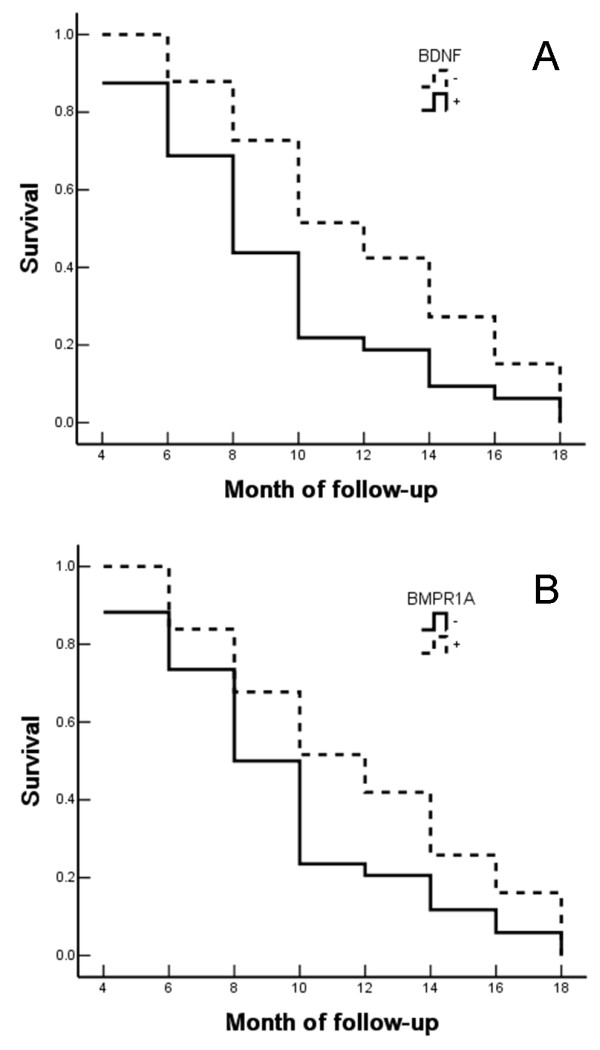
**Relationship between brain-derived neurotrophic factor and bone morphogenetic protein receptor type IA expression and survival in patients with gallbladder adenocarcinoma. (A,B)** Kaplan–Meier plots of the disease-specific survival of patients with gallbladder adenocarcinomaand **(A)** BDNF-positive and BDNF-negative expression or **(B)** BMPR1A-positive and BMPR1A-negative expression.

The relevance of positive BDNF and BMPR1A expression to patient survival was examined by univariate Kaplan–Meier survival analysis. The relevance of disease-specific survival (DSS) to the clinicopathologic characteristics was as follows.DSS was positively associated with tumor pathological type (*P* = 0.030), tumor diameter (*P* = 0.003), lymph-node metastasis (*P* = 0.005), invasion of surrounding tissues (*P* = 0.002), and T stage (*P* = 0.031). DSS was inversely associated with positive expression of BDNF (*P* = 0.013) (Table 3, Figure 4), but positively correlated with positive expression of BMPR1A (*P* = 0.042) (Table 3, Figure 4). Multivariate Cox regression survival analysis indicated that tumor maximum diameter of 20 mm or more, lymph-node metastasis, and invasion to surrounding tissue, followed by BDNF-positive expression or BMPR1A-negative expression were the most significant predictors of short DSS (Table 4).

**Table 3:** Relationships between brain-derived neurotrophic factor (BDNF) and bone morphogenetic protein receptor type IA (BMPR1A) expression and clinicopathologic characteristics and average survival of patients with gallbladder adenocarcinoma

**Clinicopathologic characteristics**	**Cases, n**	**Average survival, months**	***P***
Gender			
Male	18	10.0 (4 to 16)	0.910
Female	47	10.0 (4 to 18)	
Age, years			
≤ 45	11	8.0 (4 to 14)	0.121
> 45	54	10.0 (4 to 18)	
Pathological type			
Well differentiated	27	11.2 (4 to 18)	0.03
Moderately differentiated	20	10.0 (4 to 18)	
Poorly differentiated	12	8.0 (4 to 10)	
Mucinous adenocarcinoma	6	10.0 (6 to 16)	
Maximum, diameter of mass, mm			
< 20	19	14.0 (4 to 18)	0.003
≥ 20	46	8.0 (4 to 18)	
Metastasis to lymph node			
No	35	12.0 (4 to 18)	0.005
Yes	30	8.0 (4 to 18)	
Invasion to surrounding tissue			
No	38	10.0 (4 to 18)	0.002
Yes	27	8.0 (4 to 16)	
T stage			
T1	14	12.6 (6 to 18)	0.031
T2	23	10.7 (4 to 18)	
T3	19	8.0 (4 to 18)	
T4	9	6.2 (4 to 10)	
Gallstones			
No	29	10.9 (4 to 18)	0.135
Yes	36	9.8 (4 to 18)	
BDNF			
Positive	32	9.1 (4 to 18)	0.013
Negative	33	11.9 (6 to 18)	
BMPR1A			
Positive	31	11.7 (6 to 18)	0.042
Negative	34	9.5 (4 to 18)	

**Table 4:** Multivariate Cox regression analysis of disease-specific survival in 65 patients who underwent surgical resection of gallbladder carcinoma

**Group**	**Category**	**B**	**SE (B)**	**Exp (B)**	***P***	**95% CI for Exp (B)**	
						**Inferior**	**Superior**
Pathology type	Well/moderately/poorly differentiated carcinoma/mucous carcinoma	0.621	0.359	1.861	0.084	0.92	3.76
Tumor diameter	<20/20 mm	1.127	0.411	3.086	0.006	1.38	6.91
Lymph-node metastasis	No/yes	1.421	0.482	4.141	0.003	1.61	10.65
Invasion to surrounding tissue	No/yes	1.120	0.385	3.065	0.004	1.44	6.52
Surgery	Radical/ palliative	1.340	0.457	3.819	0.003	1.66	9.35
BDNF	Positive/negative	1.406	0.439	4.080	0.001	1.73	9.65
BMPR1A	Negative/positive	−0.885	0.389	0.413	0.023	0.19	0.89

Abbreviations: BDNF, brain-derived neurotrophic factor; BMPR1A bone morphogenetic protein receptor type IA; Exp, exponential; SE, standard error. B in SPSS means regression coefficient. SE (B) means Standard error B. Exp(B) in SPSS means Hazard ratio, also called relative risk.

## Discussion

In the current study, we found that increased expression of BDNF or decreased expression of BMPR1A were independent predictors of poor DSS rates in gallbladder adenocarcinoma. These findings suggest that these two factors might provide a novel evaluation method for determining the outcomes of various gallbladder tumor types.

Previous studies have shown high levels of BDNF and its receptor tropomyosin-related kinase B (TrkB) in various cancer types, including carcinomas of the ovary [18], colon, rectum [19], liver [20], mammary [21], cervix [5], bladder [21], chorion [22] and prostate [23]. However, the precise role of BDNF in the pathogenesis of cancer has not been fully explored. BDNF expression was reported to be significantly associated with tumor-progression factors such as poor differentiation, advanced tumor stage, and lymph-node invasion [24,25]. BDNF expression directly enhances the proliferation, survival, migratory capability, and invasiveness of tumor cells. Studies on head and neck squamous cell carcinoma (HNSCC) cell lines showed that *in vitro* stimulation with BDNF upregulated the migration and invasion of HNSCC cells, and both transient and stable suppression of TrkB resulted in significant abrogation of both constitutive and ligand-mediated migration and invasion. Using an *in vivo* mouse model of HNSCC, it was shown that downregulation of TrkB suppresses tumor growth. In human breast cancer, there was substantially greater BDNF expression within neoplastic cells compared with normal mammary epithelial cells. Similar to the current study, higher BDNF expression was significantly associated with nodal positivity, local recurrence, death from cancer, and poor overall prognosis [7]. These data suggest that BDNF may have a crucial function in mediating tumor relapse, metastasis, anti-apoptosis, and chemotherapeutic resistance in the various diseases mentioned above, and the involvement of BDNF in key cancer-related pathways makes it an attractive target for molecular targeted therapy [26,27].

BMPs are multifunctional signaling molecules that belong to the TGF-β superfamily. They were first identified based on their ability to form bone at extra skeletal sites. Later, it became evident that these bone-inducing factors also play crucial roles during development, regulating cell proliferation, differentiation, and apoptosis [27]. BMPs signal by binding to two separate (type I and II) transmembrane serine/threonine kinase receptors [28]. Three type I and type II receptors specifically bind BMP ligands. Type I receptors consist of BMPR1A (also known as ALK-3), BMPR1B (ALK-6), and activin A receptor type I (ACVR1; also known as ALK-2). However, the signaling mechanisms activated by these three receptor subtypes are not well known. We speculate that the various effects of BMPs on proliferation, differentiation, and apoptosis of tumor cells may be related to the expression levels of BMPR1A. Several studies have shown that BMPR1A plays a role in suppression of tumor progression [29,30]. In the current study, we found that BMPR1A levels were lower in gallbladder adenocarcinoma compared with benign samples. Similar to these findings, a previous study reported that benign prostate specimens expressed high levels of BMPR1A; however, prostate-cancer specimens expressed much lower levels of BMPR1A, which suggests that loss of BMPR1A may play a vital role during the progression of prostate cancer [15]. The conditions juvenile polyposis syndrome (JPS) and hereditary mixed polyposis syndrome (HMPS) are associated with an increased risk of colorectal carcinoma. Mutations in the *BMPR1A* gene have been identified in up to 20% of patients with JPS [31,32], and HMPS has also been associated with mutations in the *BMPR1A* gene [33]. BMPR1A has also been reported to be a tumor suppressor in skin tumorigenesis [16]. However, it should be noted that contradictory findings were found for squamous cell carcinoma of the lower lip, where strong expression of BMPR1A showed a significant association with advanced clinical staging and high malignancy score [34]. The current study indicated that decreased expression of BMPR1A contributes to metastasis and poor prognosis in gallbladder cancer.

Therefore, we found in the current study that positivity rates of were significantly higher for BDNF and significantly lower for BMPR1A in gallbladder adenocarcinoma compared with peritumoral adenomatous polyp, and chronic cholecystitis tissues. These findings indicate that BDNF and BMPR1A may be independent tumor markers reflecting the genesis, progression, biological behavior, and prognosis of gallbladder adenocarcinoma. More extensive studies are required to determine the mechanistic roles of BDNF and BMPR1A in the development and progression of gallbladder cancer.

Ortega *et al*. showed that BDNF induced expression of BMP7in embryonic neurons, induced the early differentiation of radial glia into glial precursors and astrocytes, and impaired neuronal migration [35]. Xu *et al*. showed that BMP7 signaling via BMPR1A and BMPR1B inhibited the proliferation of pulmonary large carcinoma cell NCI-H460 [36]. We suggest that there may be a relationship between BDNF and BMPR1A, but it remains unclear how BDNF and BMPR1A together affect the action of tumors, such as gallbladder cancer.

BDNF and its receptor TrkB have recently emerged as anticancer targets. De Farias *et al*. found increased BDNF levels in colorectal cancer tumor samples [37]. The inhibitory effect of cetuximab on cell proliferation and survival was counteracted by the addition of human recombinant BDNF. Thus, it is possible that targeting trkB could potentiate the anticancer effects of cetuximab therapy [37]. Bleuming *et al*. reported that BMP signaling via BMPR1A suppresses tumorigenesis at gastric epithelial transition zones [29]. We suggest that the findings from the current study could also be used to develop personalized therapeutic methods for gallbladder cancer based on our findings.

## Conclusion

In the current study, increased expression of BDNF and decreased expression of BMPR1A were associated with increased risk of metastasis, regional invasion, and mortality in gall bladder carcinoma. These markers might be useful as novel indicators of gallbladder adenocarcinoma outcome, assisting in the development of personalized therapies.

## Competing interests

The authors declare that they have no competing interests.

## Authors’ contributions

XM and ZY conceived of and designed the study. LX and XD were involved in recruiting patients, collecting specimens, and completing the manuscript. YW carried out the statistical analysis and was also involved in all of the experimental work. ZY prepared the pathologic sections and carried out the immunohistochemical experiments. All authors helped to draft the manuscript and all authors read and approved this final version.
